# Unraveling the complex role of *MAPT*-containing H1 and H2 haplotypes in neurodegenerative diseases

**DOI:** 10.1186/s13024-024-00731-x

**Published:** 2024-05-29

**Authors:** Chiara Pedicone, Sarah A. Weitzman, Alan E. Renton, Alison M. Goate

**Affiliations:** 1 Department of Genetics and Genomic Sciences, Icahn School of Medicine at Mount Sinai, New York, NY USA; 2 Ronald M. Loeb Center for Alzheimer’s Disease, Icahn School of Medicine at Mount Sinai, New York, NY USA; 3 Nash Family Department of Neuroscience, Icahn School of Medicine at Mount Sinai, New York, NY USA

**Keywords:** 17q21.31, Haplotypes, Inversion, Neurodegeneration, SNV, CNV, MAPT, Tau

## Abstract

A ~ 1 Mb inversion polymorphism exists within the 17q21.31 locus of the human genome as direct (H1) and inverted (H2) haplotype clades. This inversion region demonstrates high linkage disequilibrium, but the frequency of each haplotype differs across ancestries. While the H1 haplotype exists in all populations and shows a normal pattern of genetic variability and recombination, the H2 haplotype is enriched in European ancestry populations, is less frequent in African ancestry populations, and nearly absent in East Asian ancestry populations. H1 is a known risk factor for several neurodegenerative diseases, and has been associated with many other traits, suggesting its importance in cellular phenotypes of the brain and entire body. Conversely, H2 is protective for these diseases, but is associated with predisposition to recurrent microdeletion syndromes and neurodevelopmental disorders such as autism. Many single nucleotide variants and copy number variants define H1/H2 haplotypes and sub-haplotypes, but identifying the causal variant(s) for specific diseases and phenotypes is complex due to the extended linkage equilibrium. In this review, we assess the current knowledge of this inversion region regarding genomic structure, gene expression, cellular phenotypes, and disease association. We discuss recent discoveries and challenges, evaluate gaps in knowledge, and highlight the importance of understanding the effect of the 17q21.31 haplotypes to promote advances in precision medicine and drug discovery for several diseases.

**Graphical Abstract:**

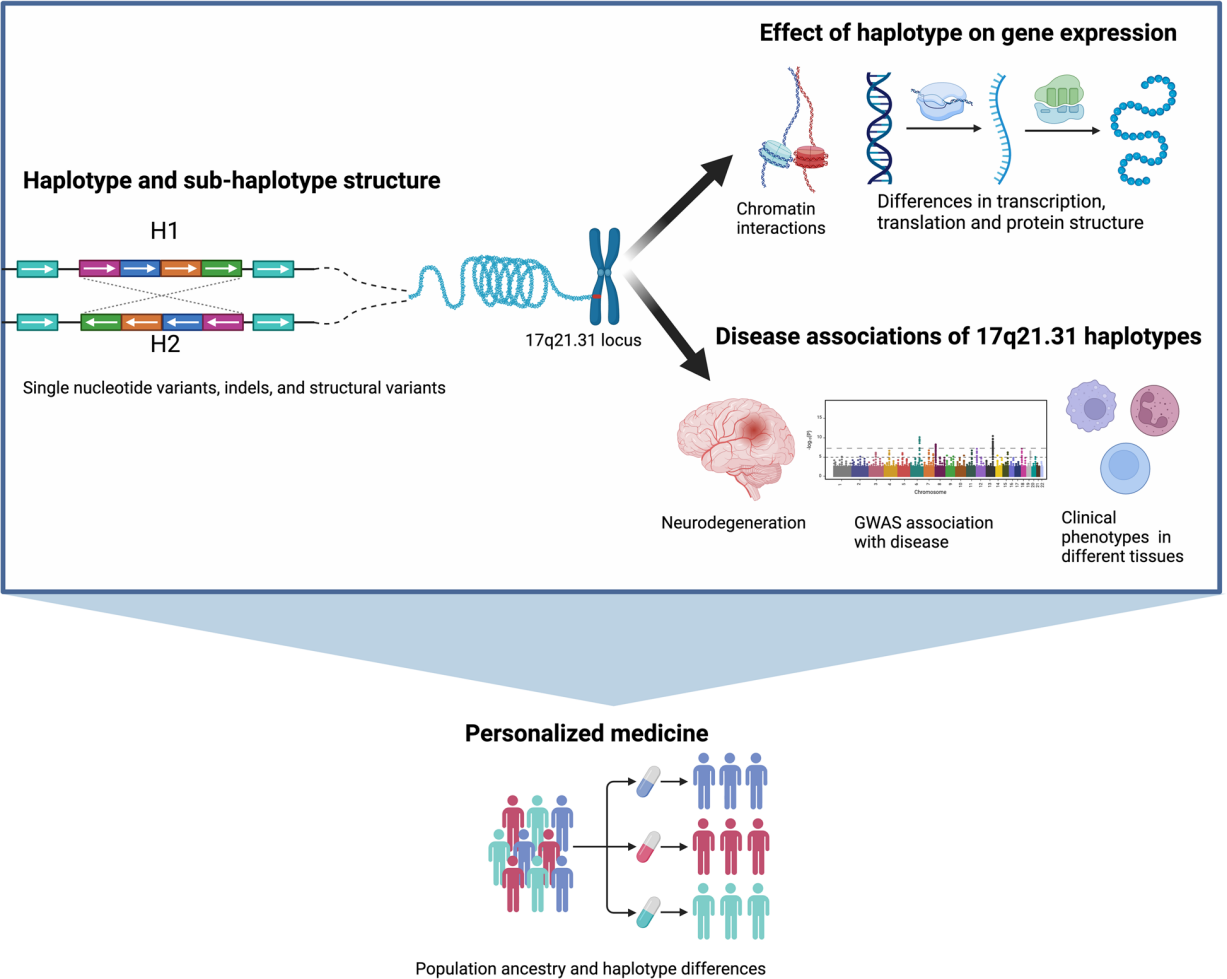

## Background

Chromosome 17 has an unusual structure that has undergone extensive intrachromosomal rearrangement resulting in a high density of segmental duplications which are low copy repeats of identical sequences [2]. Non-allelic homologous recombination along with microdeletion and inversion defines the 17q21.31 locus as a structurally complex and evolutionarily dynamic region of the genome [3]. Previous studies have shown that the locus contains two major haplotype groups (clades), H1 and H2, due to a large inversion polymorphism of ~ 1 Mb [4]. These two haplotypes have distinct patterns of linkage disequilibrium (LD), reflecting their inversion status in the 17q21.31 region [5]. H1 is in the direct orientation as defined by Zody et al. and the human reference assemblies, while H2 is in the inverted orientation, resulting in no recombination between the two haplotypes over a region of ~ 1.5 Mb [2].

The inversion contains 15 genes as well as several non-coding RNAs and pseudogenes. In the 500 Kb flanking the inversion there are 22 additional genes. It is possible that both the genes within the inversion and those flanking the inversion could demonstrate haplotype-associated differences in gene expression and regulation, resulting from altered local chromatin structure. Notably, the microtubule associated protein tau gene (*MAPT*), which codes for the homonymous protein, is contained within the inversion polymorphism [4], and as a result, this inversion locus is often referred to as the “*MAPT* haplotype”. The tau protein is important for axonal microtubule stability, neuronal survival and maturation [6, 7], and several mutations in this protein have been associated with autosomal dominant frontotemporal dementia [8]. Other genes in this region may also play a role in neurodegenerative diseases. *LRRC37A/2* exhibits copy number variation between H1 and H2, leading to an expression quantitative trait locus (eQTL) and altered astrocytic function in Parkinson’s disease (PD) [9, 10]. *KANSL1*, which also shows copy number variation and an eQTL across H1 and H2, has been linked to regulation of PINK1-dependent mitophagy in dopaminergic neurons, potentially influencing PD risk [11, 12].

The H1 haplotype is a risk factor for several neurodegenerative disorders such as Alzheimer’s disease (AD), PD, frontotemporal lobar degeneration (FTLD) [13], and primary tauopathies such as progressive supranuclear palsy (PSP) and corticobasal degeneration (CBD) [14]. It remains unclear if these traits are associated with the H1 haplotype due to alteration of the same genetic drivers or distinct causal variants or genes. It is hard to identify specific causal variants in this region because of the high LD, resulting in thousands of variants that show one hundred percent correlation with each other. In contrast, the H2 haplotype has been associated with recurrent deletions or duplications in 17q21.31 microdeletion syndrome [2] and neurodevelopmental disorders e.g., autism spectrum disorder [15].

**Fig. 1:**
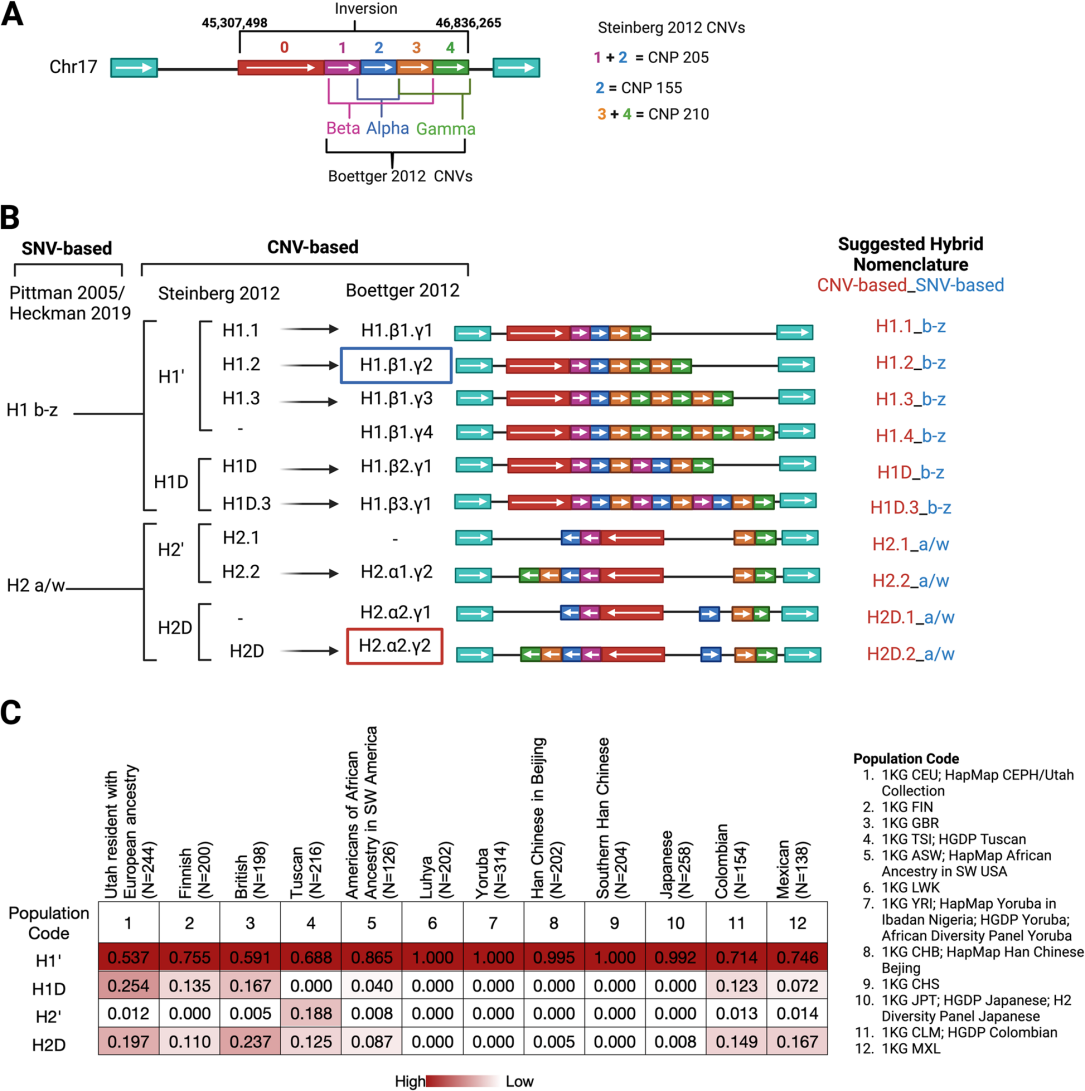
17q21.31 H1/H2 haplotype and sub-haplotype structures. **A** Structure of 17q21.31 locus with GRCh38 coordinates. Inversion locus containing color coded region blocks (0–4) based on CNVs or CNPs defined by Boettger et al. (α,β,γ) [17] or Steinberg et al. [18], respectively. **B** Haplotypes and sub-haplotypes based on SNVs or CNV repeats for H1 and H2, with suggested hybrid nomenclature using Steinberg CNV-based nomenclature merged with Pittman SNV-based nomenclature separated by underscore. GRCh38 reference genome haplotypes are highlighted in a blue or red box for H1.,β1.γ2 and H2.α2.γ2, respectively. **C** Heatmap showing frequencies of the four major sub-haplotypes in 12 populations that are composed of data from HapMap, 1000 Genomes, HGDP, African Diversity Panel, and H2 Diversity Panel defined by Steinberg et al. [18], as specified in population code list

H1 is the most common haplotype in all populations, but its frequency and the frequency of H1 sub-haplotypes differ across genetic ancestry groups. The H2 haplotype is most common in populations of European ancestry (1 KG-EUR-like populations) (0.1–0.4), and exhibits lower frequency among East Asian (1 KG-EAS-like) (0–0.09) and African (1 KG-AFR-like) (0.1–0.15) ancestry populations (Fig. 1C) [16, 17]. These population descriptors based on NASEM best practices are listed in Fig. 1C and further explained in List of Abbreviations using Population Descriptors in Genetics and Genomics Research.

**Fig. 2:**
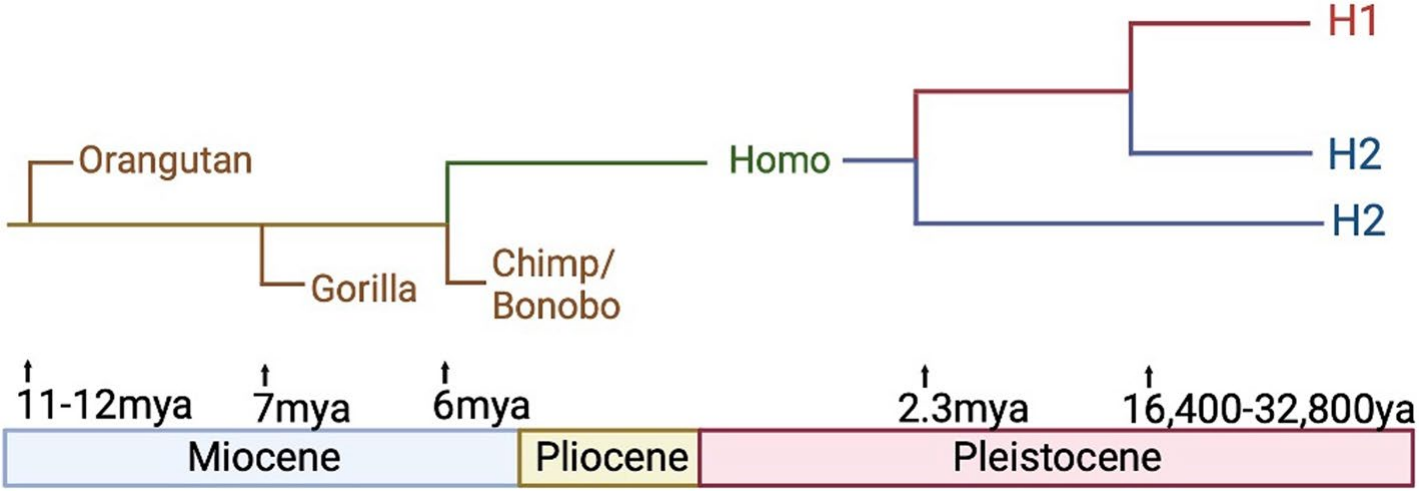
Phylogenetic Tree of 17q21.31 locus inversions and locus structure in different ancestries. **A** Phylogenetic tree summarizing findings from Donnelly et al. [20] and Zody et al. [2] regarding inversion occurrence

While the factors underlying the global H2 frequency patterns are unknown, Zody et al. suggest that an elevated H2 frequency in 1 KG-EUR-like populations might be due to selection or founder effects [2]. The 17q21.31 locus is highly polymorphic, indicating that many genetic events may have occurred throughout evolution, contributing to the complexity of this locus in all populations [19]. Donnelly et al. suggest that the most recent common ancestor (MRCA) of these haplotypes emerged in Africa or Southwest Asia 16,400–32,800 years ago [20]. Overall, these and other studies suggest that the inversion occurred twice during human evolution (Fig. 2) [2, 20]. The ancestral haplotype in great apes has an H2-like orientation, and the human lineage diverged from great apes 6 million years ago. The first inversion event is estimated to have occurred 2.3 million years ago (Mya) in the Pliocene (5.3–2.6 Mya), leading to the H1 haplotype. A more recent inversion event likely occurred in the *Homo* lineage, resulting in the H2 haplotype. Thus, if we define the haplotype based on the *Homo sapiens* reference genome, H1 is in the direct orientation while H2 is in the inverted orientation. However, the definition of ancestral orientation depends on which Homo species we define as ancestor, which is a subject of controversy. Zody et al. define ancestral as the early species *Hominini* in the Miocene (23–5.3 Mya), which carried the H2 haplotype, [2] while Donnelly et al. define ancestral as the *Homo erectus* MRCA in the Pleistocene (2.58 Mya-11,700 ya), which carried the H1 haplotype (Fig. 2) [20]. Recent studies have suggested that the H1 to H2 inversion most likely emerged originally in Europe, specifically during late Paleolithic or early Neolithic (Holocene) and spread to Asia and Africa during population expansions and migrations [21]. Further support for the European origin of H2 comes from the work of Alves et al. showing that North African populations carry H2 alleles on non-1 KG-AFR-like chromosomes [22]. The H2 haplotype is rare in 1 KG-EAS-like populations, but H1-linked segmental duplications at the *NSF* locus are found at higher frequency, as described below and shown in Fig. 1 [18].

In addition to the 17q21.31 inversion, other large inversions exist in the human genome and have been studied by fluorescence in situ hybridization (FISH) [2], polymerase chain reaction (PCR), and inverse PCR [3]. Inversion status has been shown to affect recombination, and large inversions, including at the 17q21.31 locus, share many phenotypic consequences in humans including increased risk of neurodegenerative diseases, hemophilia A [23], mental disorders [24] and autoimmune disease [25]. Some studies have shown that inversions influence local gene expression, including 17q21.31 and 8p23.1 [3, 25].

### Haplotype and sub-haplotype structure

The 17q21.31 locus harbors complex haplotypes that go beyond the structural inversion. Meiotic crossover is not possible between H1 and H2, leading to further divergence and accumulation of unique single nucleotide variants (SNVs) [18]. To date, over 2,366 SNVs have been used to define haplotypes [26] and sub-haplotypes of H1/H2 [27–29]. The most commonly used SNVs to define the H1/H2 haplotype clades are rs8070723 [18] and rs1052553 [20], while sub-haplotype definitions are often based on rs1467967, rs242557, rs3785883, rs2471738, and rs7521 [29]. Homologous recombination following the inversion has led to duplications and microdeletions, further enriching subtyping of the H1 and H2 haplotype clades. Several studies have attempted to untangle the haplotype and sub-haplotype structures (Fig. 1A & B). However, these SNV- and copy number variant (CNV)-based classification schemes do not fully overlap and therefore should be considered separately [26]. In this review, we suggest a new nomenclature that integrates existing CNV- and SNV-based nomenclatures separated by an underscore, for example Steinberg 2012-defined CNV sub-haplotype H1.1 combined with Pittman 2005-defined SNV sub-haplotype b would be called H1.1_b (Fig. 1B).

The locus structure was first described by Stefansson et al., with inversion breakpoints defined at chr17:43,628,944–44,571,603 (GRCh37–hg19 reference assembly) [16]. Boettger and colleagues described in detail how the two haplotypes may have diverged, accumulating duplications and deletions to create several sub-haplotypes [17]. In this model, they divided the 3’ end of the locus into three inversion repeats (GRCh37–hg19): α (chr17:46,135,415–46,289,349), β (chr17:46,087,894–46356512), γ (chr17:46,289,349–46,489,400). However, this partitioning method does not provide the correct definition for H1D [18] and has only a partial duplication of the β repeat. Thus, subdivision of copy number variation within the locus into four regions (1,2,3,4) allows for better definition of partial duplication events seen in H1D as region 1 and 2, as shown in Fig. 1. A sub-classification of the haplotypes proposed by Steinberg et al. [18] defined two repeat loci in the 3’ end segment of the inversion polymorphism: “*KANSL1* short duplication” (copy number polymorphism 155 [CNP155]) and “*KANSL1* long duplication” (CNP205) that both span the gene promoter and exon 1; and “*NSF* duplication” (CNP210) that spans exon 10 of *NSF*. Connecting these findings with Boettger et al., the CNP210 repeat is the γ repeat (regions 3 and 4), while CNP155 and CNP205 are α (region 2) and part of β (regions 1 and 2), respectively. Interestingly, α duplication is found in the H2D haplotype while β duplication is found in the H1D haplotype, resulting in two different *KANSL1* gene duplications in the two haplotypes [18]. Duplication and retention of the non-inverted α fragment defines H2D based on many SNVs in the γ region, likely due to divergence of the sub-haplotypes (Table 1) [18]. Alongside segmental duplication, the inversion locus also exhibits microdeletion with loss of a 424 Kb region encompassing six genes (chr17:45,613,580–46037741; GRCh38–hg38) [30], or smaller deletions, describing a critical 160 Kb genomic fragment encompassing *MAPT, STH*, and *KIAA1267* associated with developmental delay and facial dysmorphism [31].

**Table 1:** 17q21.31 haplotype- and sub-haplotype-defining SNVs. SNV alleles observed on: GRCh38 direct orientation haplotype H1 or sub-haplotype H1.2_b (GRCh38-NC_000017.11); GRCh38 direct orientation sub-haplotype H1.2_e (GRCh38-NT_187663.1); GRCh38 inverted orientation H2.1 (from Steinberg et al. [18], no reference assembly was included in the original paper); and GRCh38 inverted orientation haplotype H2 or sub-haplotype H2D (GRCh38-NT_167251.2). SNV genomic coordinates and annotated gene are shown. Amino acid positions are based on the following isoform references: *CRHR1*: NP_001289945.1, *SPPL2C*: NP_787078.2, *MAPT*: NP_058519.3, *STH*: NP_001007533.1, *KANSL1*: NP_001180395.1, *NSF*: NP_006169.2. Bold text denotes non-synonymous SNVs predicted to cause amino acid substitutions

SNV	Gene	Consequence	Hg38, Chr17	H1.2_b	H1.2_e	H1.2_c	H2.1	H2D	Reference(s)
rs241039	LINC02210-CRHR1	-	45,637,307	A				T	[20]
rs393152	LINC02210-CRHR1	-	45,641,777	A				G	[32]
rs434428	LINC02210-CRHR1	-	45,648,318	G				A	[20]
rs241027	LINC02210-CRHR1	-	45,658,112	A				G	[20]
rs2049515	LINC02210-CRHR1	-	45,684,490	C				T	[20]
rs10491144	LINC02210-CRHR1	-	45,695,758	A				C	[20]
rs10514879	LINC02210-CRHR1	-	45,725,605	C				T	[20]
rs2902662	LINC02210-CRHR1	-	45,729,559	G				A	[20]
rs17563599	LINC02210-CRHR1	-	45,730,589	A				C	[18]
rs11079718	LINC02210-CRHR1	-	45,762,585	A				T	[20]
rs1396862	CRHR1	intron variant	45,825,631	G				A	[20]
**rs16940681**	**CRHR1**	**E280Q**	**45,834,793**	**G**				**C**	[33]
**rs62621252**	**SPPL2C**	**S224P**	**45,845,576**	**T**				**C**	[33]
**rs62054815**^**a**^	**SPPL2C**	**A332T**	**45,845,900**	**G**				**A**	[33]
**rs12185233**^**a**^	**SPPL2C**	**R461P**	**45,846,288**	**G**				**C**	[33]
**rs12185268**^**a**^	**SPPL2C**	**I471V**	**45,846,317**	**A**				**G**	[33]
**rs12373123**^**a**^	**SPPL2C**	**S601P**	**45,846,707**	**T**				**C**	[33]
**rs12373139**^**a**^	**SPPL2C**	**G620R**	**45,846,764**	**G**				**A**	[33]
**rs12373142**	**SPPL2C**	**P643R**	**45,846,834**	**C**				**G**	[33]
rs1078830	MAPT-AS1	-	45,868,746	T				C	[20]
rs916793	MAPT-AS1	-	45,877,320	G				A	[20]
rs1467967^b^	MAPT	intron variant	45,908,813	G	A	A		A	[29, 34]
rs17563986	MAPT	intron variant	45,913,906	A				G	[18]
rs17649553	MAPT	intron variant	45,917,282	C				T	[35]
rs242557^b^	MAPT	intron variant	45,942,346	G	G	A		G	[27]
rs17650901	MAPT	5' UTR variant	45,962,325	A				G	[20]
rs1800547	MAPT	intron variant	45,974,480	A				G	[16]
rs17651213	MAPT	intron variant	45,974,558	G				A	[20]
rs3785883^b^	MAPT	intron variant	45,977,067	A	G	G		G	[29, 34]
rs1981997	MAPT	intron variant	45,979,401	G				A	[18]
**rs63750417**	**MAPT**	**P202L**	**45,983,409**	**C**				**T**	[33]
**rs62063786**	**MAPT**	**D285N**	**45,983,657**	**G**				**A**	[33]
**rs62063787**	**MAPT**	**V289A**	**45,983,670**	**T**				**C**	[33]
**rs17651549**	**MAPT**	**R370W**	**45,983,912**	**C**				**T**	[33]
**rs10445337**	**MAPT**	**S447P**	**45,990,034**	**T**				**C**	[33]
rs1052553	MAPT	P544P	45,996,523	A				G	[20]
rs2471738^b^	MAPT	intron variant	45,998,697	C	C	T		C	[27]
**rs62063857**	**STH**	**Q7R**	**45,999,299**	**A**				**G**	[33]
rs8070723	MAPT	intron variant	46,003,698	A				G	[18, 36, 37]
rs9468	MAPT	3'UTR variant	46,024,197	T				C	[16, 38]
rs7521^b^	MAPT	3'UTR variant	46,028,029	A	A	G		G	[29, 34]
**rs34579536**	**KANSL1**	**I1085T**	**46,031,540**	**A**				**G**	[33]
**rs36076725**	**KANSL1**	**F917L**	**46,033,166**	**G**				**A**	[11]
**rs35833914**	**KANSL1**	**D914E**	**46,033,175**	**G**				**A**	[11]
**rs34043286**	**KANSL1**	**S718P**	**46,039,753**	**A**				**G**	[33]
rs12150447	KANSL1	intron variant	46,050,759	A				C	[20]
rs2838	KANSL1	intron variant	46,063,981	A				G	[20]
rs1468241	KANSL1	intron variant	46,118,787	A				G	[20]
rs1528075	KANSL1	intron variant	46,143,088	T				G	[20]
rs1528072	KANSL1	intron variant	46,159,359	C				A	[20]
**rs1881193**	**KANSL1**	**R247S**	**46,171,403**	**T**				**C**	[33]
rs2732703	ARL17B and LRRC37A	intron variants	46,275,856	T				G	[39]
rs2957297	ARL17B and LRRC37A	intron variants	46,290,846	C				G	[18]
rs199457^c^	LRRC37A2 and NSF	intron variants	46,718,103	C			C	T	[18]
rs199456^c^	LRRC37A2 and NSF	intron variants	46,720,553	C			C	T	[18]
rs199451^c^	LRRC37A2 and NSF	intron variants	46,724,418	G			G	A	[18]
rs199448^c^	LRRC37A2 and NSF	intron variants	46,731,635	A			A	G	[18]
rs199533^c^	NSF	K702K	46,751,565	G			G	A	[18]

^a^ region containing the two hESC H3K4me1 enhancers

^b^ SNV identifying H1 sub-haplotype

^c^ SNV identifying H2 sub-haplotypes

**Fig. 3:**
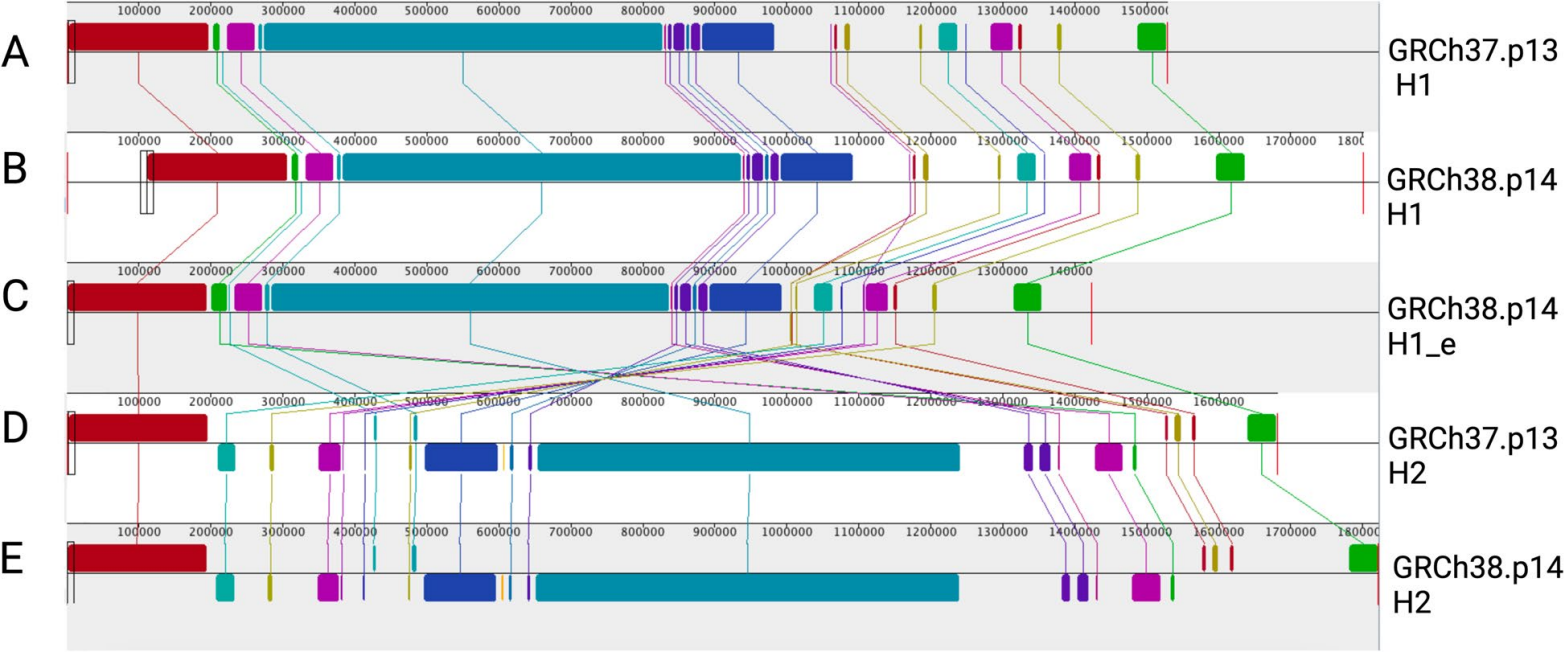
17q21.31 inversion structure. Alignment with Mauve showing inversion of H1 and H2 assemblies in GRCh37 and GRCh38 with 22 Local Collinear Blocks (LCBs) and LCB weight of 4119. H1 assemblies: **A** GRCh37.p13 scaffold NC_000017.10, **B** GRCh38.p14 scaffold NC_000017.11. Alternative H1 assembly: **C** GRCh38 scaffold NT_187663.1. H2 assemblies: **D** GRCh37 scaffold NT_167251.1 (GL00258.1), **E** GRCh38 Scaffold NT_167251.2 (GL00258.2)

The 17q21.31 locus has two alternate scaffolds for the H2 haplotype: chr17_ctg5_hap1/GL00025.1 in the GRCh37 assembly and GL00025.2 in the GRCh38 assembly, of which both were constructed based on 1 KG-EUR-like individuals. The H1 haplotype is present in the GRCh38 assembly as H1.2_b and can be found with an alternative sub-haplotype reference NT_187663.1/KI270908v1_alt in GRCh38, H1.2_c. Although the breakpoints of the inversion are not shown, there is observed homology in the first and last 200,000 base pairs of the H2 assembly with respect to the H1 assembly for GRCh37 and GRCh38, as seen in Fig. 3 [4]. The LD within each of the haplotypes results in the inheritance of all variants as a single unit, known as a haploblock. This complicates the process of fine-mapping and identifying disease-causal variants that are independent of the inversion and CNVs. As a result of the structural complexity in this region, SNVs on GWAS arrays often fail the Hardy Weinberg equilibrium test, resulting in exclusion of these variants during quality assessments, leading to large gaps devoid of SNVs flanking the inversion in GWAS data [40]. Despite the availability of two alternate assemblies in GRCh38 and one in GRCh37, the 17q21.31 haplotype structures in diverse ancestries remain poorly understood. The lack of a specific backbone for sub-haplotypes may contribute to underestimation of genetic differences between them in duplicated and truncated genes. Moreover, these assemblies do not provide a proper reference for populations with different ancestries, which could lead to inaccuracies in SNV representation and ultimately misrepresentation of haplotypes. For example, while 1 KG-EAS-like populations have a higher rate of gene duplication, they have an extremely low frequency of the H2 haplotype (Fig. 1C) [18]. Additionally, without a reference for 1 KG-AFR-like populations, the definition of the H2 haplotype based solely on genotyping array data may be questionable if variant frequencies and LD patterns differ by ancestry. These limitations underscore the importance of considering ancestry when characterizing the 17q21.31 locus and emphasize the need to generate ancestry-specific references with the novel long read sequencing data and tools available. The recent release of the pangenome, encompassing 47 diverse human genomes, offers a renewed perspective to study the locus in terms of different structural variants, ancestries, and SNVs [41].

The definitions of haplotypes and sub-haplotypes in this region have long been based on SNV characterization [18, 20, 26–28]. While the use of TaqMan SNV genotyping of a few SNVs for haplotype definition may lead to inaccurate answers, the screening arrays (GDA or GSA) can be used to define sub-haplotypes and ancestry. Although it is rarely used, FISH with probes specific to the H1 or H2 haplotype [2, 18] can be used to identify inverted haplotypes. To address the need for high-throughput screening methods, several techniques have been developed such as digital droplet PCR (ddPCR) [42] and 3D PCR [10], respectively allowing identification of the inversion and sub-haplotypes defined by different CNVs. Puig et al. demonstrate the presence of inversion repeats on both sides of the inversion with ddPCR, showing the efficiency of these methods and promising scalability for clinical testing [42].

Overall, the 17q21.31 locus is a complex and dynamic genomic region where inversion, SNVs, segmental duplication, and microdeletion likely impact gene expression. Thus, further studies are needed to better understand the genetic variation within this region, and how it ultimately affects human health and disease across global populations.

### Effect of haplotype on gene expression

Chromosomal rearrangement observed in the 17q21.31 locus may drive gene expression changes in the region, although the effects are largely unexplored or inconsistent across studies, as is the case for *MAPT* expression [5, 10, 28, 43]. No studies to date have described the effect of the inversion on the expression of genes surrounding the ~ 1 Mb inversion or globally. It is likely that the inversion and CNVs result in altered chromatin structure, leading to changes in gene regulation and expression. Thus, cell type-specific chromosome accessibility and chromatin interaction data across the region will be important to determine which genes in which cell type(s) show altered expression between the different haplotypes.

Pivotal changes in gene function and expression may be driven by missense variants causing protein coding changes or synonymous variants affecting RNA stability and splicing. Table 1 summarizes the haplotype- and sub-haplotype-defining SNVs defining and the effects of known variants at the protein level [11, 33] Several missense variants with differing alleles were previously reported between H1 and H2 by Campoy et al. and others, and in this review, we report two more in *KANSL1* rs36076725 (Asp to Glu) and rs35833914 (Phe to Leu). Overall, there are 19 missense variants in five genes within the inversion (*CRHR1, SPPL2C, MAPT, STH, KANSL1*). Five of these are conservative changes, while 14 are non-conservative. Of the latter, two gains of proline and four losses of proline substitutions may affect protein structure, stability, or function, along with polar to non-polar changes [44]**.**

**Fig. 4:**
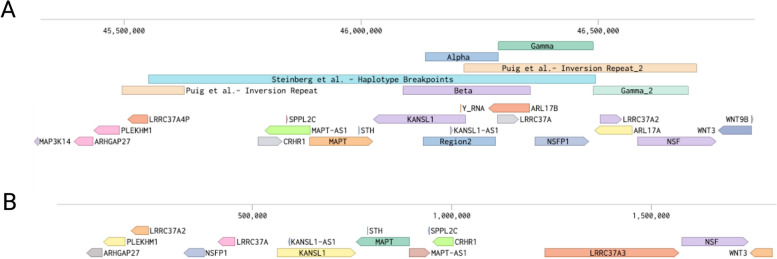
Genes in H1 and H2 haplotypes. Annotated genes in (**A**) H1 Chr17 assembly GRCh38-NC_000017.11-chr17 (45,309,498–46836264) and (**B**) H2 GRCh38-NT_167251.2 (1–1,821,992) with Benching from NCBI Refseq Annotation GCF_000001405.40-RS_2023_03

Inversions and CNVs can influence chromatin structure, potentially altering locus accessibility to transcription factors, thereby enhancing or repressing transcription. For example, two hESC H3K4me1 super enhancers chr17:45,844,803–45,845,787 (985 bp) and chr17:45,845,788–45,846,771 (984 bp) are present in the H1 haplotype. However, only the first enhancer, containing a CTCF regulatory element (chr17:45,844,748–45,844,929), is annotated in the H2 haplotype. There is a need for further investigation to determine whether this enhancer, which regulates stem cell proliferation, is truly absent in the H2 haplotype or if there is an oversight in the annotation [45]. Furthermore, the presence of duplications and deletions alter gene copy numbers, subsequently impacting protein levels. Figure 4 shows several genes in the inversion region annotated on GRCh38.

Genes at the breakpoint of the inversion may be affected by changes in enhancer or chromatin accessibility. Of the genes at the 5’ breakpoint of the inversion, two regulate lysosomal and autophagosome functions: the Rho GTPase activating protein *ARHGAP27* [46] and the Rubicon Homology (RH) domain containing *PLEKHM1*. They respectively regulate vesicular trafficking through Rac1/Cdc42 [47] and lysosomal functions interacting with Rab7 [48]. Massive Parallel Reporter Assays (MPRA) nominated *PLEKHM1* as a putative causal gene in PSP, which was further validated with CRISPRi in an iPSC-derived cell system [49]. Interestingly, lysosome dysfunction is a common feature of multiple neurodegenerative diseases [50]. The NF-kB inducing kinase *MAP3K14* is present on the 5’ end of the inversion, and is known to regulate non-canonical NF-kB signaling in immunity [51], another dysregulated pathway in many neurodegenerative diseases. Lastly, *WNT3* is present on the 3’ end of the inversion, which is essential for neuronal patterning through β-catenin signaling [52].

Genes present in loci with copy number variation can have duplications or deletions that can change their expression. In this locus *KANSL1, NSF, ARL17,* and *LRRC37A* are genes with important functions that have been linked to neurodegeneration. *KAT8 Regulatory NSL Complex Subunit 1 (KANSL1)* encodes the lysine acetyltransferase KAT8 that plays a role in the histone deacetylase complex, driving chromatin modifications as part of the NSL complex [53]. Haploinsufficiency in this gene phenocopies the microdeletion phenotype with developmental delay [54]. However, it is still unknown if the truncated duplications in *KANSL1* [18] containing exon 1–3 (CNP155) or exon 1–4 (CNP210) code for functional proteins as they do not contain key amino acids necessary for KAT8 activity (residues 850–882) [18]. Moreover, these truncated proteins may act as a dominant negative altering function of full length KAT8, possibly leading to genome-wide differences in histone acetylation and gene expression. *KANSL1* regulates autophagy and mitophagy through transcriptional regulation of STXStx in mice [55]. This function is impaired in individuals with one copy of *KANSL1* suggesting haploinsufficiency, which could be rescued by a small molecule autophagy activator: 13-cis RA. Loss of function variants in exon 2 of *KANSL1* (e.g. G306*) are known to cause Koolen De Vries syndrome (KdVS) [30, 56], but variants in exon 8 [57] identified on the H2 haplotype have not been associated with neurodevelopmental disorders. *N-ethylmaleimide sensitive factor* (*NSF*) encodes a vesicle fusing ATPase that catalyzes vesicle transport within the Golgi, essential for synaptic vesicle turnover. γ duplications increase transcript levels of the pseudogene *NSFP1* that contains the first 13 exons of *NSF*. Although the effect on protein stability is still unknown, the CNVs in this locus are associated with cocaine dependence [58].

*ADP-ribosylation factor-like protein 17* (*ARL17*) and *Leucine-Rich Repeat-Containing Protein 37A* (*LRRC37A*) are present in the 5’ end of the γ region. Higher γ repeat number increases expression of full-length protein isoforms from these gene families that exhibit highly conserved structure. The functions of *ARL17A* and *ARL17B* remain unknown [59], while the *LRRC37A* family includes three genes (*LRRC37A*, *LRRC37A2*, *LRRC37A3*) and the pseudogene *LRRC37A4* which is only present on the H1 haplotype [5]. CNVs of *LRRC37A2*, observed on the H2 haplotype and on some H1 sub-haplotypes, are associated with reduced risk of PD and have been linked to increased astrocytic migration and chemotaxis [10].

Lastly, two genes in the inversion, *CRHR1* and *MAPT*, are not located in copy number variation regions 1–4, but their duplication is correlated with neurological diseases [15, 60]. *CRHR1* encodes the corticotropin releasing hormone receptor. This protein is essential for regulation of the hypothalamic–pituitary–adrenal pathway, and its duplication causes autistic spectrum disorder [15]. The *MAPT* gene encodes the tau protein, which is highly expressed in neurons and known to stabilize axonal microtubules. *MAPT* mutations increase tau aggregation, destabilizing microtubules [4, 61–63], and have been linked to autosomal dominant frontotemporal dementia (FTD) [64, 65]. The 439-kb microduplication at the 17q21.31 locus, associated with neurological diseases encompasses *MAPT, IMP5, CRHR1*, and *STH* [66, 67]. While *MAPT* duplications have also been associated with disease, the roles of the other genes still warrant investigation [60, 68]. Additionally, a rare missense variant in *MAPT* (A152T) has been associated with increased risk for several neurodegenerative diseases, including AD, FTD, and progressive primary aphasia, highlighting the complex genetic landscape of neurodegeneration [69, 70].

Several studies have reported a possible role of the H1/H2 haplotypes in regulating *MAPT*, however, the reported effects on expression are inconsistent between studies [4, 5, 28]. The H1 haplotype has higher *MAPT* expression compared to the H2 haplotype in blood [5] and in temporal cortex and cerebellum of AD subjects [4]. However, this is not the case for brain samples from healthy individuals [28]. Furthermore, allele specific expression has shown increased *MAPT* promoter strength in the H1 haplotype and increased exon 10 retention and 4R/3R ratio [43]. However, this effect may be due to the contribution of the H1_c sub-haplotype (rs242557) [28] which is associated with higher risk for tauopathies [29]. Functional variants within the H1 haplotype have been identified that disrupt a ribosomal binding site in *MAPT*, potentially affecting its translation [71]. This finding highlights the complexity of the haplotype’s impact on *MAPT* across multiple regulatory levels, from transcriptional to post-transcriptional modifications. The inconsistencies between studies may reflect cell type-dependent effects on splicing and expression in the brain, and the cell type heterogeneity that is altered in neurodegenerative diseases. Highly characterized iPSC-derived models and single cell sequencing analysis in the human brain could help resolve these contradictions.

**Fig. 5:**
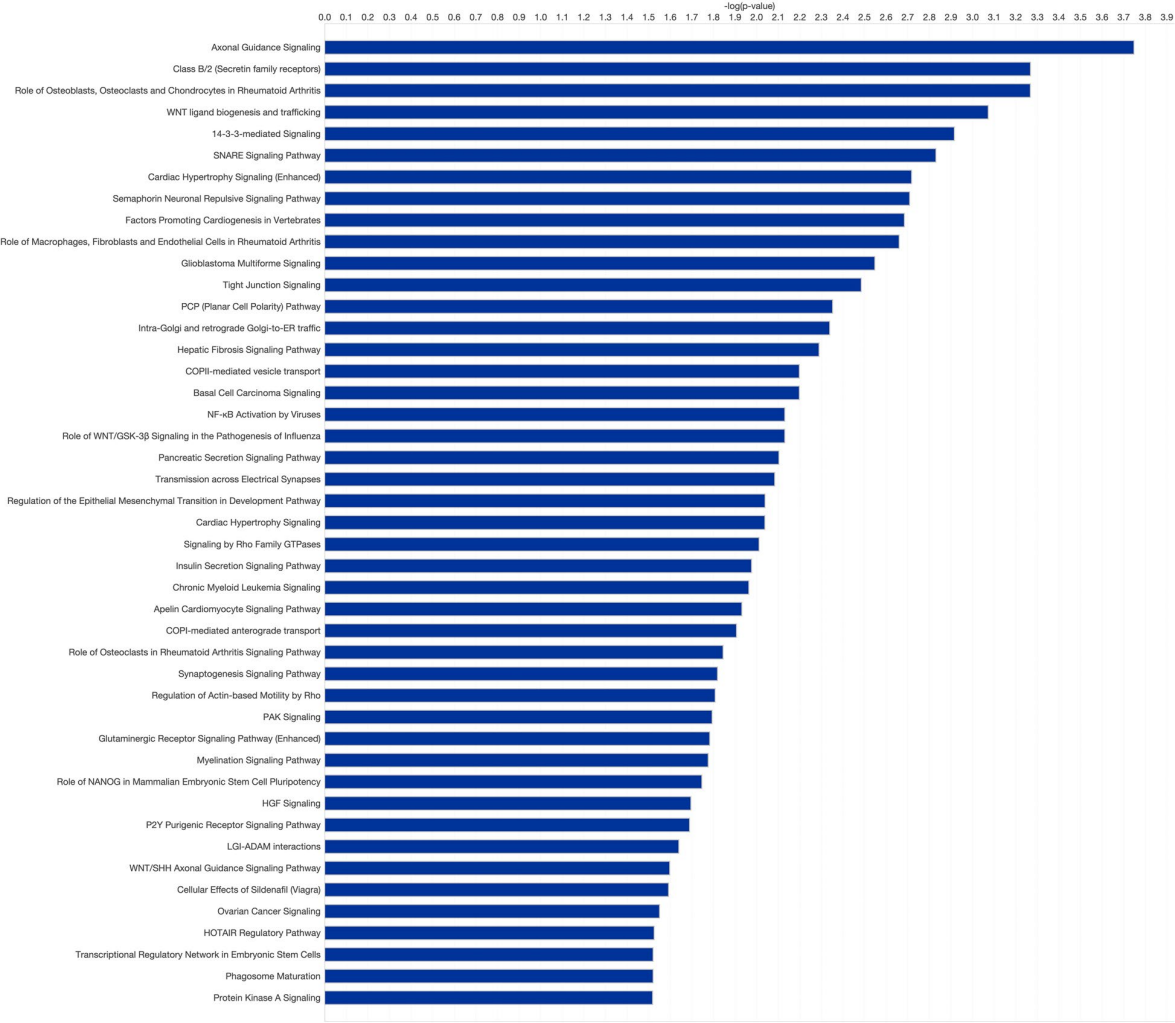
Pathway analysis for genes in 17q21.31 haplotype region. Enriched pathways derived from Ingenuity Pathway Analysis (IPA) based on the genes in and around the inversion

In Fig. 5**,** we highlight a 17q21.31 locus top canonical pathway analysis. Brain gene expression for genes present within the 17q21.31 locus 1 Mb inversion region in addition to 1 Mb surrounding the inversion were compared for H1 and H2 homozygous individuals using Ingenuity Pathway Analysis (IPA) and Ensembl 110 to identify the top canonical pathways exhibiting differential gene expression across the haplotypes. Significant pathways include axonal guidance, synaptogenesis, tight junction signaling, NF-kB activation by viruses, cardiomyocytes hypertrophic signaling, and pathways shared by macrophages, fibroblasts, and endothelial cells in Rheumatoid arthritis. Based on our pathway analysis (Fig. 5), the stem cell pluripotency pathway and NANOG are altered between the H1 and H2 haplotypes, supporting the hypothesis that loss of the hESC H3K4me1 super enhancer may modulate this effect. Our pathway analysis highlights how this complex locus can differentially influence pathways in a cell type-dependent manner, creating complex cell-intrinsic and -extrinsic effects that ultimately impact several organs and diseases. Epigenetic studies in this locus have shown that methylation regulates differential expression [72]. Furthermore, chromatin accessibility and chromatin looping could also be altered by haplotype-dependent differences in chromosomal structure, altering transcription by displacing enhancers or repressors. Supporting evidence for this hypothesis comes from recent data showing that cis-regulatory elements (cCREs) for *MAPT*, inside and outside the haplotype breakpoints, define possible cell type-dependent differential regulation [73].

#### Disease associations of 17q21.31 haplotypes

**Fig. 6:**
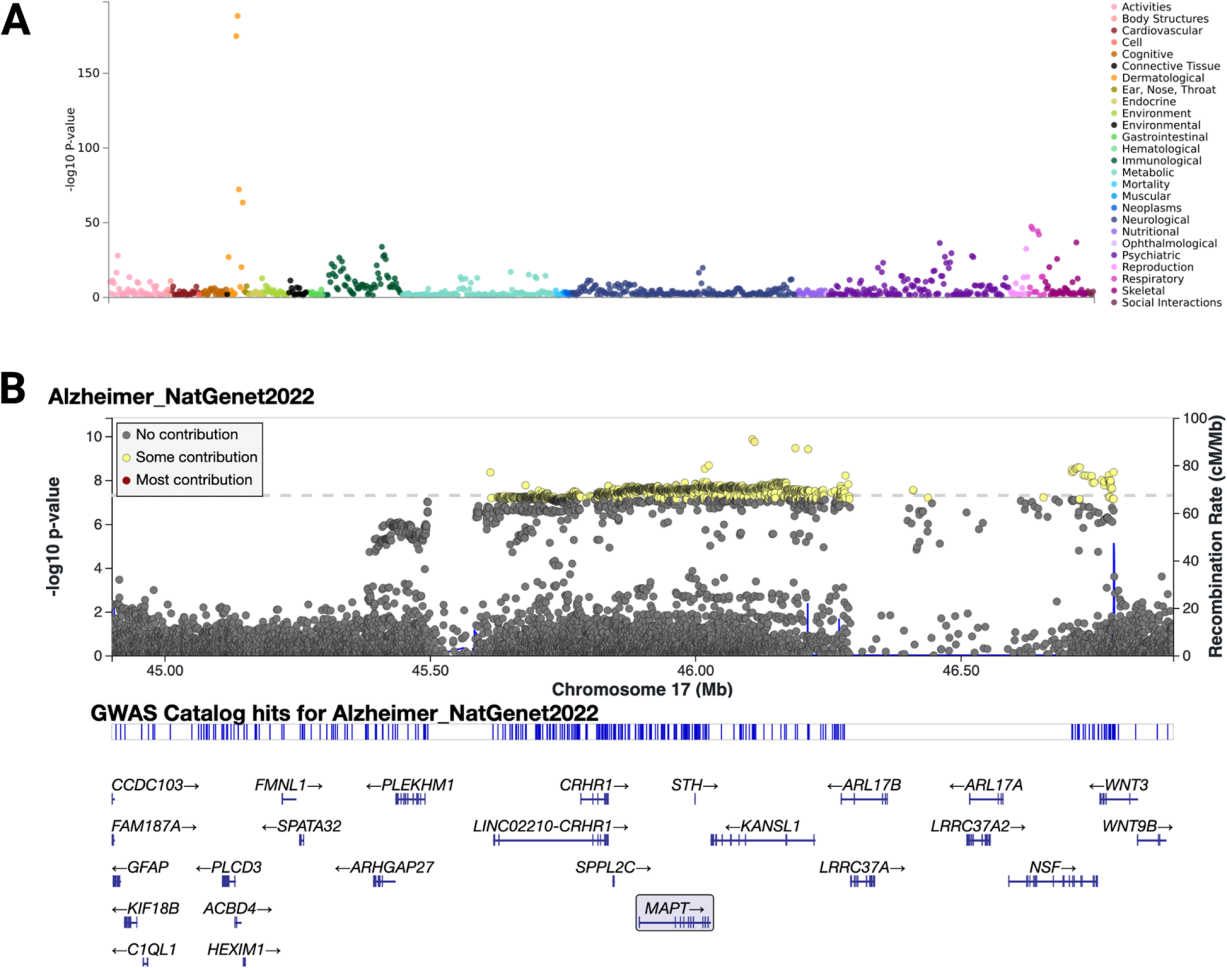
17q21.31 trait association. **A** Traits associated with 17q21.31 haplotypes based on rs1052553 SNV from https://atlas.ctglab.nl/PheWAS. **B** Bellenguez et al. [74] AD GWAS Locus Zoom showing the 17q21.31 haplotype region

Multiple studies have associated the 17q21.31 haplotypes with phenotypic differences in health and disease. The H1 haplotype is a risk factor for several neurodegenerative diseases, exhibiting striking odds ratios (ORs) for PSP (OR = 5.46 for H1 relative to H2; OR = 1.91 for H1c relative to other H1 sub-haplotypes) [36, 37] and CBD (OR = 3.7) [32] risk, with lower odds ratios for AD (OR = 1.06) [74], PD (OR = 1.30) [35, 75], and behavioral variant FTD (bvFTD) (OR = 1.2) [76] risk (Table 2). Moreover, specific H1 sub-haplotypes have been associated with lower risk for PD (OR = 0.77) [10] (Fig. 6). The stronger associations with PSP and CBD compared to AD and PD suggest that the H1 haplotype may play a central role in clinical and neuropathological features that differ between these illnesses, and that the variants or gene(s) in this region influencing these diseases may be different or at least overlapping but distinct. One possible explanation for this increased risk in PSP and CBD might be through higher regional vulnerability of H1 haplotype neurons in the midbrain, a pivotal region for PSP and CBD pathology. A challenge in dissecting these differences has been the lack of an animal model, given these haplotype differences are unique to humans. However, as human induced pluripotent stem cell models improve, it will be possible to test the effects of these haplotypes in both in vitro and xenotransplantation models. Overlapping significant loci for PSP, CBD, and FTD highlight *NSF* and the *MAPT* exonic rs199533 SNV as being consistently correlated with increased risk in these diseases [14]. Furthermore, Pastor et al. report the H1e haplotype to be present in 16% of PSP patients but not in controls, suggesting that SNVs in *CRHR1, IMP3, MAPT* or *STH* may be risk factors for PSP [38]. Conversely, the H2 haplotype has been previously associated with the 17q21.31 microdeletion syndrome [31, 56, 77] and autism [2]. Several other microduplications have been found in 17q21.31 causing developmental delay, microcephaly, or autistic spectrum disorders, but it remains unclear how they connect to the inversion [15, 78].

**Table 2:** 17q21.31 haplotype- and sub-haplotype-defining SNVs associated with neurodegenerative diseases. Odds ratios and p-values are reported for the H1-linked risk allele of each listed SNV

Disease	SNV based Haplotype	Odds Ratio	p-value	Reference
AD	rs199515	1.06	9.3 × 10^-13	[74]
	rs2732703	1.36	6.4 × 10^-7	[39]
PD	rs393152	1.30	1.95 × 10^-16	[79]
	rs199533^a^	1.28	1.09 × 10^-14	[79]
	rs17649553	1.30	2.37 × 10^-48	[35]
	rs199502	1.31	7.35 × 10^-22	[75]
CBD	rs393152, rs242557^b^	3.7	1.42 × 10^-12	[32]
PSP	rs8070723	5.46	1.5 × 10^-116	[36]
	rs242557^b^	1.91	1.58 × 10^-22	[37]
bvFTD	rs8070723	1.2	3.14 × 10^-3	[76]

^a^Alternate protective allele is H2D

^b^ Risk allele is H1_c

While the association of the 17q21.31 haplotypes with various neurodegenerative diseases is well documented, influence of the locus also extends into more nuanced areas of disease progression and phenotype expression, particularly in PD and related cognitive impairments. Recent large-scale GWAS focusing on PD age at onset (AAO) did not identify significant associations with the 17q21.31 locus [80, 81]. This suggests that while the H1 haplotype is implicated in disease risk, it does not appear to play a role influencing the timing of disease onset in PD. Similarly, comprehensive analyses in PD, Parkinson’s disease dementia (PDD), and dementia with Lewy bodies (DLB) have not detected association signals at 17q21.31, underscoring the locus’ complex relationship with cognitive impairment in these conditions [82]. Contrasting these findings, candidate gene analyses and smaller studies have identified potential associations with clinical disease progression, highlighting the nuanced influence of the 17q21.31 locus on neurodegenerative phenotypes [83, 84]. These disparities may reflect the limitations of underpowered studies or true differences in the genetics underlying these related phenotypes and emphasize the need for robust, large-scale research to elucidate the genetic modifiers of PD, cognitive impairment, and dementia.

In a broader context, associations with the H1 and H2 haplotypes encompass a wide range of phenotypes including dermatological, immunologic, respiratory, psychiatric, and social traits (Fig. 6A). There are also connections with various cellular differences such as blood counts and anthropometric parameters [33]. While cellular phenotypes have not been explored, clinical traits and susceptibility to diseases highlight differences in the immune system and hormonal balance. H2 haplotype carriers exhibit increased numbers of erythrocytes, basophils, and neutrophils, as well as reduced leucocyte and eosinophil count [33]. Studies focusing on H2 homozygous 1 KG-EUR-like individuals have reported sub-clinical changes in cortical surface area [85], blood pressure, lung function, and bone mineral density, with increased susceptibility to depression, chronic obstructive pulmonary disease (COPD), osteoarthritis, and autoimmune diseases [33]. Furthermore, sex-dependent differences have been observed and found to be dependent on hormonal balance. In H2 carrier females, higher progesterone levels may raise fertility rates [16] and increase risk for ovarian cancer [33]. In H2 carrier males higher testosterone levels are associated with later puberty and androgenetic alopecia (AGA), also known as male pattern baldness (MPB), the most common type of hair loss in humans [33]. Moreover, a meta-analysis in 2012 revealed an association between AGA and the 17q21.31 locus, and later studies showed tau expression in hair follicles [86]. In contrast to the findings by Campoy et al., an analysis of the 23andMe cohort, with a larger sample size than Campoy et al., found an increased risk of Parkinson’s disease among individuals with AGA compared to unaffected controls, particularly among those aged over 70 years [33]. In conclusion, 17q21.31 haplotypes play vital roles in both health and disease, influencing a myriad of clinical traits and disease phenotypes.

## Conclusions

The 17q21.31 locus is characterized by a complex structure, stemming from inversion events, CNVs, and SNVs. The existing classification systems using SNV- and CNV-based nomenclature remain disconnected, motivating a unified approach to discern haplotype identification within the locus. Due to the Eurocentric bias in biomedical science, current studies and reference datasets primarily focus on 1 KG-EUR-like individuals. It is imperative that 17q21.31 haplotype and sub-haplotype structures are extensively investigated across different ancestries and admixed populations to truly elucidate locus structure diversity. Additionally, the H1 haplotype has been associated with neurodegenerative diseases primarily in 1 KG-EUR-like populations. It is critical to investigate these associations in other populations, to determine how many are driven by the comparison of H1 to H2 or by association with H1 sub-haplotypes in non-1 KG-EUR-like populations, where H2 is less frequent or absent. For example, in 1 KG-AFR-like individuals the H2 haplotype is not protective for PD [87]. The impact of H1 and H2 haplotypes on gene regulation and expression in specific brain cell types has not been comprehensively examined beyond bulk RNA-seq using brain homogenates. iPSC-derived models serve as effective tools for studying diseases associated with *MAPT* mutations in both 2D cultures of neurons [88–91] and organoids [9]. These models have also been used to understand the impact of CNVs within this locus [92]. Generation of iPSC CRISPR edited isogenic lines with the whole ~ 1 Mb inversion is challenging due to the size of the locus and unclear breakpoints. Thus, current studies require larger sample sizes than isogenic comparison to overcome donor effects and should be considered observational studies. Moreover, two mouse models have been developed to study local haplotypes: one carrying a *KANSL1* microdeletion [93, 94] and the other containing a 190 Kb fragment of the haplotype that includes *SPPLC2* and *MAPT* in the H1 or H2 orientation [71]. A limitation of these models is that they only contain short fragments of the inversion, thus providing only a partial understanding of these specific genes rather than the extended locus. Comprehensive analysis is needed to capture the complex interplay between the inversion, CNVs, and SNVs, as haplotype effects may be driven by these elements acting in concert. Tools such as MPRA and CRISPRi screening can enable the identification of causal SNVs linked to this locus and associated diseases, similar to the role of *PLEKHM1* in PSP [49]. Fully understanding the intricate interrelationships within this locus is essential to pave the way for developing advanced tools to decipher predisposition to several neurodegenerative diseases, thereby ushering in a new era of precision medicine that improves diagnosis and bolsters prevention and treatment modalities. An enhanced understanding of the 17q21.31 locus is critical for improving neurodegenerative disease interventions.

## Data Availability

All data analyzed during this study are available from the authors on request.

## Abbreviations

LD: Linkage disequilibrium
SNVs: Single nucleotide variants
CNVs: Copy number variants
MAPT: Microtubule Associated Protein Tau gene
eQTL: Expression quantitative trait locus
PD: Parkinson’s disease
AD: Alzheimer’s disease
FTLD: Frontotemporal lobar degeneration
PSP: Progressive supranuclear palsy
CBD: Corticobasal degeneration
MRCA: Most recent common ancestor
Mya: Million years ago
PCR: Polymerase chain reaction
FISH: Fluorescence In Situ Hybridization
ddPCR: Digital droplet PCR
RH: Rubicon homology
MPRA: Massive parallel reporter assays
NSF: N-ethylmaleimide sensitive factor
ARL1: ADP-ribosylation factor-like protein 17
LRRC37: Leucine-Rich Repeat-Containing Protein 37
IPA: Ingenuity pathway analysis
STH: Saitohin
COPD: Chronic obstructive pulmonary disease
AGA: Androgenetic alopecia
MPB: Male pattern baldness
OR: Odds ratio

## Glossary

Haplotype: A haplotype refers to a set of DNA alleles along a single chromosome that tend to be inherited together.
LD: In population genetics, linkage disequilibrium (LD) is the non-random association of alleles at different loci in a given population. Loci are said to be in linkage disequilibrium when the frequency of association of their different alleles is higher than expected if the loci were independent and associated randomly.
CNVs: Copy number variants (CNVs) refers to a class of genetic polymorphism in which genomic sequences are repeated, and the number of repeats varies between individuals of the same species.
eQTL: An expression quantitative trait locus (eQTL) explains a fraction of the genetic variance of a gene expression phenotype. Standard eQTL analysis involves direct association testing between markers of genetic variation and gene expression levels typically measured in hundreds or thousands of individuals.
MPRA: A massively parallel reporter assay (MPRA) can functionally validate thousands of regulatory elements simultaneously using high-throughput sequencing and barcode technology.

## References

[1] National Academies of Sciences E, Medicine (2023). Using Population Descriptors in Genetics and Genomics Research: A New Framework for an Evolving Field.

[2] Zody MC, Jiang Z, Fung HC, Antonacci F, Hillier LW, Cardone MF, Graves TA, Kidd JM, Cheng Z, Abouelleil A (2008). Evolutionary toggling of the MAPT 17q21.31 inversion region. Nat Genet.

[3] Giner-Delgado C, Villatoro S, Lerga-Jaso J, Gayà-Vidal M, Oliva M, Castellano D, Pantano L, Bitarello BD, Izquierdo D, Noguera I (2019). Evolutionary and functional impact of common polymorphic inversions in the human genome. Nat Commun.

[4] Allen M, Kachadoorian M, Quicksall Z, Zou F, Chai HS, Younkin C, Crook JE, Pankratz VS, Carrasquillo MM, Krishnan S (2014). Association of MAPT haplotypes with Alzheimer’s disease risk and MAPT brain gene expression levels. Alzheimers Res Ther.

[5] de Jong S, Chepelev I, Janson E, Strengman E, van den Berg LH, Veldink JH, Ophoff RA (2012). Common inversion polymorphism at 17q21.31 affects expression of multiple genes in tissue-specific manner. BMC Genomics.

[6] Dawson HN, Ferreira A, Eyster MV, Ghoshal N, Binder LI, Vitek MP (2001). Inhibition of neuronal maturation in primary hippocampal neurons from tau deficient mice. J Cell Sci.

[7] Kadavath H, Hofele RV, Biernat J, Kumar S, Tepper K, Urlaub H, Mandelkow E, Zweckstetter M (2015). Tau stabilizes microtubules by binding at the interface between tubulin heterodimers. Proc Natl Acad Sci U S A.

[8] Strang KH, Golde TE, Giasson BI (2019). MAPT mutations, tauopathy, and mechanisms of neurodegeneration. Lab Invest.

[9] Bowles KR, Silva MC, Whitney K, Bertucci T, Berlind JE, Lai JD, Garza JC, Boles NC, Mahali S, Strang KH (2021). ELAVL4, splicing, and glutamatergic dysfunction precede neuron loss in MAPT mutation cerebral organoids. Cell.

[10] Bowles KR, Pugh DA, Liu Y, Patel T, Renton AE, Bandres-Ciga S, Gan-Or Z, Heutink P, Siitonen A, Bertelsen S (2022). 17q21.31 sub-haplotypes underlying H1-associated risk for Parkinson’s disease are associated with LRRC37A/2 expression in astrocytes. Molecular Neurodegeneration.

[11] Soto-Beasley AI, Walton RL, Valentino RR, Hook PW, Labbé C, Heckman MG, Johnson PW, Goff LA, Uitti RJ, McLean PJ (2020). Screening non-MAPT genes of the Chr17q21 H1 haplotype in Parkinson's disease. Parkinsonism Relat Disord.

[12] Soutar MPM, Melandri D, O'Callaghan B, Annuario E, Monaghan AE, Welsh NJ, D'Sa K, Guelfi S, Zhang D, Pittman A (2022). Regulation of mitophagy by the NSL complex underlies genetic risk for Parkinson's disease at 16q11.2 and MAPT H1 loci. Brain..

[13] Park HK, Chung SJ (2013). New perspective on parkinsonism in frontotemporal lobar degeneration. J Mov Disord.

[14] Yokoyama JS, Karch CM, Fan CC, Bonham LW, Kouri N, Ross OA, Rademakers R, Kim J, Wang Y, Höglinger GU (2017). Shared genetic risk between corticobasal degeneration, progressive supranuclear palsy, and frontotemporal dementia. Acta Neuropathol.

[15] Grisart B, Willatt L, Destrée A, Fryns JP, Rack K, de Ravel T, Rosenfeld J, Vermeesch JR, Verellen-Dumoulin C, Sandford R (2009). 17q21.31 microduplication patients are characterised by behavioural problems and poor social interaction. J Med Genet..

[16] Stefansson H, Helgason A, Thorleifsson G, Steinthorsdottir V, Masson G, Barnard J, Baker A, Jonasdottir A, Ingason A, Gudnadottir VG (2005). A common inversion under selection in Europeans. Nat Genet.

[17] Boettger LM, Handsaker RE, Zody MC, McCarroll SA (2012). Structural haplotypes and recent evolution of the human 17q21.31 region. Nat Genet..

[18] Steinberg K, Antonacci F, Sudmant P, Kidd J, Campbell C, Vives L, Malig M, Scheinfeldt L, Beggs W, Ibrahim M (2012). Structural Diversity and African Origin of the 17q21.31 Inversion Polymorphism. Nature genetics.

[19] Harerimana NV, Goate AM, Bowles KR (2022). The influence of 17q21.31 and APOE genetic ancestry on neurodegenerative disease risk. Front Aging Neurosci.

[20] Donnelly MP, Paschou P, Grigorenko E, Gurwitz D, Mehdi SQ, Kajuna SL, Barta C, Kungulilo S, Karoma NJ, Lu RB (2010). The distribution and most recent common ancestor of the 17q21 inversion in humans. Am J Hum Genet.

[21] Espinosa I, Alfonso-Sánchez MA, Gómez-Pérez L, Peña JA (2023). Neolithic expansion and the 17q21.31 inversion in Iberia: an evolutionary approach to H2 haplotype distribution in the Near East and Europe. Mol Genet Genomics.

[22] Alves JM, Lima AC, Pais IA, Amir N, Celestino R, Piras G, Monne M, Comas D, Heutink P, Chikhi L (2015). Reassessing the Evolutionary History of the 17q21 Inversion Polymorphism. Genome Biol Evol.

[23] Lakich D, Kazazian HH, Antonarakis SE, Gitschier J (1993). Inversions disrupting the factor VIII gene are a common cause of severe haemophilia A. Nat Genet.

[24] Okbay A, Baselmans BM, De Neve JE, Turley P, Nivard MG, Fontana MA, Meddens SF, Linnér RK, Rietveld CA, Derringer J (2016). Genetic variants associated with subjective well-being, depressive symptoms, and neuroticism identified through genome-wide analyses. Nat Genet.

[25] Salm MP, Horswell SD, Hutchison CE, Speedy HE, Yang X, Liang L, Schadt EE, Cookson WO, Wierzbicki AS, Naoumova RP, Shoulders CC (2012). The origin, global distribution, and functional impact of the human 8p23 inversion polymorphism. Genome Res.

[26] Corces MR, Shcherbina A, Kundu S, Gloudemans MJ, Frésard L, Granja JM, Louie BH, Eulalio T, Shams S, Bagdatli ST (2020). Single-cell epigenomic analyses implicate candidate causal variants at inherited risk loci for Alzheimer's and Parkinson's diseases. Nat Genet.

[27] Pittman AM, Myers AJ, Abou-Sleiman P, Fung HC, Kaleem M, Marlowe L, Duckworth J, Leung D, Williams D, Kilford L (2005). Linkage disequilibrium fine mapping and haplotype association analysis of the tau gene in progressive supranuclear palsy and corticobasal degeneration. J Med Genet.

[28] Myers AJ, Pittman AM, Zhao AS, Rohrer K, Kaleem M, Marlowe L, Lees A, Leung D, McKeith IG, Perry RH (2007). The MAPT H1c risk haplotype is associated with increased expression of tau and especially of 4 repeat containing transcripts. Neurobiol Dis.

[29] Heckman MG, Kasanuki K, Brennan RR, Labbé C, Vargas ER, Soto AI, Murray ME, Koga S, Dickson DW, Ross OA (2019). Association of MAPT H1 subhaplotypes with neuropathology of lewy body disease. Mov Disord.

[30] Koolen DA, Sharp AJ, Hurst JA, Firth HV, Knight SJ, Goldenberg A, Saugier-Veber P, Pfundt R, Vissers LE, Destrée A (2008). Clinical and molecular delineation of the 17q21.31 microdeletion syndrome. J Med Genet.

[31] Dubourg C, Sanlaville D, Doco-Fenzy M, Le Caignec C, Missirian C, Jaillard S, Schluth-Bolard C, Landais E, Boute O, Philip N (2011). Clinical and molecular characterization of 17q21.31 microdeletion syndrome in 14 French patients with mental retardation. Eur J Med Genet.

[32] Kouri N, Ross OA, Dombroski B, Younkin CS, Serie DJ, Soto-Ortolaza A, Baker M, Finch NCA, Yoon H, Kim J (2015). Genome-wide association study of corticobasal degeneration identifies risk variants shared with progressive supranuclear palsy. Nat Commun.

[33] Campoy E, Puig M, Yakymenko I, Lerga-Jaso J, Cáceres M (2022). Genomic architecture and functional effects of potential human inversion supergenes. Philos Trans R Soc Lond B Biol Sci.

[34] Wider C, Vilariño-Güell C, Jasinska-Myga B, Heckman MG, Soto-Ortolaza AI, Cobb SA, Aasly JO, Gibson JM, Lynch T, Uitti RJ (2010). Association of the MAPT locus with Parkinson’s disease. Eur J Neurol.

[35] Nalls MA, Pankratz N, Lill CM, Do CB, Hernandez DG, Saad M, DeStefano AL, Kara E, Bras J, Sharma M (2014). Large-scale meta-analysis of genome-wide association data identifies six new risk loci for Parkinson's disease. Nat Genet.

[36] Höglinger GU, Melhem NM, Dickson DW, Sleiman PM, Wang LS, Klei L, Rademakers R, de Silva R, Litvan I, Riley DE (2011). Identification of common variants influencing risk of the tauopathy progressive supranuclear palsy. Nat Genet.

[37] Chen Z, Chen JA, Shatunov A, Jones AR, Kravitz SN, Huang AY, Lawrence L, Lowe JK, Lewis CM, Payan CAM (2019). Genome-wide survey of copy number variants finds MAPT duplications in progressive supranuclear palsy. Mov Disord.

[38] Pastor P, Ezquerra M, Perez JC, Chakraverty S, Norton J, Racette BA, McKeel D, Perlmutter JS, Tolosa E, Goate AM (2004). Novel haplotypes in 17q21 are associated with progressive supranuclear palsy. Ann Neurol.

[39] Jun G, Ibrahim-Verbaas CA, Vronskaya M, Lambert JC, Chung J, Naj AC, Kunkle BW, Wang LS, Bis JC, Bellenguez C (2016). A novel Alzheimer disease locus located near the gene encoding tau protein. Mol Psychiatry.

[40] Hardy GH (1908). Mendelian Proportions in a Mixed Population. Science.

[41] Liao W-W, Asri M, Ebler J, Doerr D, Haukness M, Hickey G, Lu S, Lucas JK, Monlong J, Abel HJ (2023). A draft human pangenome reference. Nature.

[42] Puig M, Lerga-Jaso J, Giner-Delgado C, Pacheco S, Izquierdo D, Delprat A, Gayà-Vidal M, Regan JF, Karlin-Neumann G, Cáceres M (2020). Determining the impact of uncharacterized inversions in the human genome by droplet digital PCR. Genome Res.

[43] Caffrey TM, Joachim C, Paracchini S, Esiri MM, Wade-Martins R (2006). Haplotype-specific expression of exon 10 at the human MAPT locus. Hum Mol Genet.

[44] Lee DY, Kim KA, Yu YG, Kim KS (2004). Substitution of aspartic acid with glutamic acid increases the unfolding transition temperature of a protein. Biochem Biophys Res Commun.

[45] Barakat TS, Halbritter F, Zhang M, Rendeiro AF, Perenthaler E, Bock C, Chambers I (2018). Functional Dissection of the Enhancer Repertoire in Human Embryonic Stem Cells. Cell Stem Cell.

[46] Katoh Y, Katoh M (2004). Identification and characterization of ARHGAP27 gene in silico. Int J Mol Med.

[47] Sakakibara T, Nemoto Y, Nukiwa T, Takeshima H (2004). Identification and characterization of a novel Rho GTPase activating protein implicated in receptor-mediated endocytosis. FEBS Lett.

[48] McEwan DG, Popovic D, Gubas A, Terawaki S, Suzuki H, Stadel D, Coxon FP, Miranda de Stegmann D, Bhogaraju S, Maddi K (2015). PLEKHM1 regulates autophagosome-lysosome fusion through HOPS complex and LC3/GABARAP proteins. Mol Cell.

[49] Cooper YA, Teyssier N, Dräger NM, Guo Q, Davis JE, Sattler SM, Yang Z, Patel A, Wu S, Kosuri S (2022). Functional regulatory variants implicate distinct transcriptional networks in dementia. Science.

[50] Udayar V, Chen Y, Sidransky E, Jagasia R (2022). Lysosomal dysfunction in neurodegeneration: emerging concepts and methods. Trends Neurosci.

[51] Pflug KM, Sitcheran R. Targeting NF-κB-Inducing Kinase (NIK) in Immunity, Inflammation, and Cancer. Int J Mol Sci. 2020;21:8470. 10.3390/ijms21228470 PMC7696043 33187137

[52] Clements WK, Ong KG, Traver D (2009). Zebrafish wnt3 is expressed in developing neural tissue. Dev Dyn.

[53] Sheikh BN, Guhathakurta S, Akhtar A (2019). The non-specific lethal (NSL) complex at the crossroads of transcriptional control and cellular homeostasis. EMBO Rep.

[54] Zollino M, Orteschi D, Murdolo M, Lattante S, Battaglia D, Stefanini C, Mercuri E, Chiurazzi P, Neri G, Marangi G (2012). Mutations in KANSL1 cause the 17q21.31 microdeletion syndrome phenotype. Nat Genet.

[55] Li T, Lu D, Yao C, Li T, Dong H, Li Z, Xu G, Chen J, Zhang H, Yi X (2022). Kansl1 haploinsufficiency impairs autophagosome-lysosome fusion and links autophagic dysfunction with Koolen-de Vries syndrome in mice. Nat Commun.

[56] Koolen DA, Pfundt R, Linda K, Beunders G, Veenstra-Knol HE, Conta JH, Fortuna AM, Gillessen-Kaesbach G, Dugan S, Halbach S (2016). The Koolen-de Vries syndrome: a phenotypic comparison of patients with a 17q21.31 microdeletion versus a KANSL1 sequence variant. Eur J Hum Genet.

[57] Bigoni S, Marangi G, Frangella S, Panfili A, Ognibene D, Squeo GM, Merla G, Zollino M. Clinical Genetics Can Solve the Pitfalls of Genome-Wide Investigations: Lesson from Mismapping a Loss-of-Function Variant in KANSL1. Genes. 2020;11:1177. 10.3390/genes11101177 PMC7600039 33050294

[58] Cabana-Domínguez J, Roncero C, Grau-López L, Rodríguez-Cintas L, Barral C, Abad AC, Erikson G, Wineinger NE, Torrico B, Arenas C (2016). A Highly Polymorphic Copy Number Variant in the NSF Gene is Associated with Cocaine Dependence. Sci Rep.

[59] Vargová R, Wideman JG, Derelle R, Klimeš V, Kahn RA, Dacks JB, Eliáš M: A Eukaryote-Wide Perspective on the Diversity and Evolution of the ARF GTPase Protein Family. Genome Biol Evol. 2021;13:evab157. 10.1093/gbe/evab157 PMC8358228 34247240

[60] Wallon D, Bonnevalle A, Rovelet-Lecrux A, Lagarde J, Sarazin M, Bottlaender M, Sellal F, Jonveaux T, Heitz C, Magnin E (2020). Phenotypes associated with MAPT duplications. Alzheimers Dement.

[61] Barghorn S, Zheng-Fischhöfer Q, Ackmann M, Biernat J, von Bergen M, Mandelkow EM, Mandelkow E (2000). Structure, microtubule interactions, and paired helical filament aggregation by tau mutants of frontotemporal dementias. Biochemistry.

[62] Kobayashi K, Kidani T, Ujike H, Hayashi M, Ishihara T, Miyazu K, Kuroda S, Koshino Y (2003). Another phenotype of frontotemporal dementia and parkinsonism linked to chromosome-17 (FTDP-17) with a missense mutation of S305N closely resembling Pick's disease. J Neurol.

[63] Alonso Adel C, Mederlyova A, Novak M, Grundke-Iqbal I, Iqbal K (2004). Promotion of hyperphosphorylation by frontotemporal dementia tau mutations. J Biol Chem.

[64] Hutton M, Lendon CL, Rizzu P, Baker M, Froelich S, Houlden H, Pickering-Brown S, Chakraverty S, Isaacs A, Grover A (1998). Association of missense and 5'-splice-site mutations in tau with the inherited dementia FTDP-17. Nature.

[65] Ghetti B, Oblak AL, Boeve BF, Johnson KA, Dickerson BC, Goedert M (2015). Invited review: Frontotemporal dementia caused by microtubule-associated protein tau gene (MAPT) mutations: a chameleon for neuropathology and neuroimaging. Neuropathol Appl Neurobiol.

[66] Rovelet-Lecrux A, Hannequin D, Guillin O, Legallic S, Jurici S, Wallon D, Frebourg T, Campion D (2010). Frontotemporal dementia phenotype associated with MAPT gene duplication. J Alzheimers Dis.

[67] Le Guennec K, Quenez O, Nicolas G, Wallon D, Rousseau S, Richard AC, Alexander J, Paschou P, Charbonnier C, Bellenguez C (2017). 17q21.31 duplication causes prominent tau-related dementia with increased MAPT expression. Mol Psychiatry.

[68] Wallon D, Boluda S, Rovelet-Lecrux A, Thierry M, Lagarde J, Miguel L, Lecourtois M, Bonnevalle A, Sarazin M, Bottlaender M (2021). Clinical and neuropathological diversity of tauopathy in MAPT duplication carriers. Acta Neuropathol.

[69] Coppola G, Chinnathambi S, Lee JJ, Dombroski BA, Baker MC, Soto-Ortolaza AI, Lee SE, Klein E, Huang AY, Sears R (2012). Evidence for a role of the rare p.A152T variant in MAPT in increasing the risk for FTD-spectrum and Alzheimer's diseases. Hum Mol Genet.

[70] Ramos EM, Dokuru DR, Van Berlo V, Wojta K, Wang Q, Huang AY, Miller ZA, Karydas AM, Bigio EH, Rogalski E (2019). Genetic screen in a large series of patients with primary progressive aphasia. Alzheimers Dement.

[71] Simone R, Javad F, Emmett W, Wilkins OG, Almeida FL, Barahona-Torres N, Zareba-Paslawska J, Ehteramyan M, Zuccotti P, Modelska A (2021). MIR-NATs repress MAPT translation and aid proteostasis in neurodegeneration. Nature.

[72] Li Y, Chen JA, Sears RL, Gao F, Klein ED, Karydas A, Geschwind MD, Rosen HJ, Boxer AL, Guo W (2014). An Epigenetic Signature in Peripheral Blood Associated with the Haplotype on 17q21.31, a Risk Factor for Neurodegenerative Tauopathy. PLOS Genetics.

[73] Rogers BB, Anderson AG, Lauzon SN, Davis MN, Hauser RM, Roberts SC, Rodriguez-Nunez I, Trausch-Lowther K, Barinaga EA, Hall PI (2024). Neuronal <em>MAPT</em> expression is mediated by long-range interactions with <em>cis</em>-regulatory elements. The American Journal of Human Genetics.

[74] Bellenguez C, Küçükali F, Jansen IE, Kleineidam L, Moreno-Grau S, Amin N, Naj AC, Campos-Martin R, Grenier-Boley B, Andrade V (2022). New insights into the genetic etiology of Alzheimer’s disease and related dementias. Nat Genet.

[75] Nalls MA, Blauwendraat C, Vallerga CL, Heilbron K, Bandres-Ciga S, Chang D, Tan M, Kia DA, Noyce AJ, Xue A (2019). Identification of novel risk loci, causal insights, and heritable risk for Parkinson's disease: a meta-analysis of genome-wide association studies. Lancet Neurol.

[76] Ferrari R, Hernandez DG, Nalls MA, Rohrer JD, Ramasamy A, Kwok JB, Dobson-Stone C, Brooks WS, Schofield PR, Halliday GM (2014). Frontotemporal dementia and its subtypes: a genome-wide association study. Lancet Neurol.

[77] Rao PN, Li W, Vissers LE, Veltman JA, Ophoff RA (2010). Recurrent inversion events at 17q21.31 microdeletion locus are linked to the MAPT H2 haplotype. Cytogenet Genome Res.

[78] Mc Cormack A, Taylor J, Te Weehi L, Love DR, George AM (2014). A case of 17q21.31 microduplication and 7q31.33 microdeletion, associated with developmental delay, microcephaly, and mild dysmorphic features. Case Rep Genet..

[79] Simón-Sánchez J, Schulte C, Bras JM, Sharma M, Gibbs JR, Berg D, Paisan-Ruiz C, Lichtner P, Scholz SW, Hernandez DG (2009). Genome-wide association study reveals genetic risk underlying Parkinson's disease. Nat Genet.

[80] Blauwendraat C, Heilbron K, Vallerga CL, Bandres-Ciga S, von Coelln R, Pihlstrøm L, Simón-Sánchez J, Schulte C, Sharma M, Krohn L (2019). Parkinson's disease age at onset genome-wide association study: Defining heritability, genetic loci, and α-synuclein mechanisms. Mov Disord.

[81] Grover S, Kumar Sreelatha AA, Pihlstrom L, Domenighetti C, Schulte C, Sugier PE, Radivojkov-Blagojevic M, Lichtner P, Mohamed O, Portugal B (2022). Genome-wide Association and Meta-analysis of Age at Onset in Parkinson Disease: Evidence From the COURAGE-PD Consortium. Neurology.

[82] Lesley W, Raquel R, Alejandro M, Ruth C, Michael AL, Maryam S, Catherine B, Leon H, Cornelis B, Andrew BS, et al. Investigation of the genetic aetiology of Lewy body diseases with and without dementia. medRxiv. 2023:2023.2010.2017.23297157.

[83] Liu G, Peng J, Liao Z, Locascio JJ, Corvol JC, Zhu F, Dong X, Maple-Grødem J, Campbell MC, Elbaz A (2021). Genome-wide survival study identifies a novel synaptic locus and polygenic score for cognitive progression in Parkinson's disease. Nat Genet.

[84] Real R, Martinez-Carrasco A, Reynolds RH, Lawton MA, Tan MMX, Shoai M, Corvol JC, Ryten M, Bresner C, Hubbard L (2023). Association between the LRP1B and APOE loci and the development of Parkinson's disease dementia. Brain.

[85] Wang H, Makowski C, Zhang Y, Qi A, Kaufmann T, Smeland OB, Fiecas M, Yang J, Visscher PM, Chen C-H (2023). Chromosomal inversion polymorphisms shape human brain morphology. Cell Rep.

[86] Heilmann-Heimbach S, Hochfeld LM, Paus R, Nöthen MM (2016). Hunting the genes in male-pattern alopecia: how important are they, how close are we and what will they tell us?. Exp Dermatol.

[87] Okunoye O, Ojo OO, Abiodun O, Abubakar S, Achoru C, Adeniji O, Agabi O, et al. MAPT allele and haplotype frequencies in Nigerian Africans: Population distribution and association with Parkinson’s disease risk and age at onset. Parkinsonism Relat Disord. 2023;113:105517. 10.1016/j.parkreldis.2023.105517 37467655

[88] Silva MC, Cheng C, Mair W, Almeida S, Fong H, Biswas MHU, Zhang Z, Huang Y, Temple S, Coppola G (2016). Human iPSC-Derived Neuronal Model of Tau-A152T Frontotemporal Dementia Reveals Tau-Mediated Mechanisms of Neuronal Vulnerability. Stem Cell Reports.

[89] Karch CM, Kao AW, Karydas A, Onanuga K, Martinez R, Argouarch A, Wang C, Huang C, Sohn PD, Bowles KR (2019). A Comprehensive Resource for Induced Pluripotent Stem Cells from Patients with Primary Tauopathies. Stem Cell Reports.

[90] Mahali S, Martinez R, King M, Verbeck A, Harari O, Benitez BA, Horie K, Sato C, Temple S, Karch CM (2022). Defective proteostasis in induced pluripotent stem cell models of frontotemporal lobar degeneration. Transl Psychiatry.

[91] Minaya MA, Mahali S, Iyer AK, Eteleeb AM, Martinez R, Huang G, Budde J, Temple S, Nana AL, Seeley WW (2023). Conserved gene signatures shared among MAPT mutations reveal defects in calcium signaling. Front Mol Biosci.

[92] Miguel L, Rovelet-Lecrux A, Chambon P, Joly-Helas G, Rousseau S, Wallon D, Epelbaum S, Frébourg T, Campion D, Nicolas G, Lecourtois M (2022). Generation of 17q21.31 duplication iPSC-derived neurons as a model for primary tauopathies. Stem Cell Res.

[93] Arbogast T, Iacono G, Chevalier C, Afinowi NO, Houbaert X, van Eede MC, Laliberte C, Birling M-C, Linda K, Meziane H (2017). Mouse models of 17q21.31 microdeletion and microduplication syndromes highlight the importance of Kansl1 for cognition. PLOS Genetics.

[94] Iacono G, Benevento M, Dubos A, Herault Y, van Bokhoven H, Nadif Kasri N, Stunnenberg HG (2017). Integrated transcriptional analysis unveils the dynamics of cellular differentiation in the developing mouse hippocampus. Sci Rep.

